# Types of Injuries and the Severity of Shoulder Dysfunction Associated with Diabetes Mellitus in Patients with Functional Impairment: A Case–Control Study

**DOI:** 10.3390/biomedicines12112634

**Published:** 2024-11-18

**Authors:** Mercedes Fuentes-Murguia, Karla B. Carrazco-Peña, Osiris G. Delgado-Enciso, Joel Castellanos-Gomez, Gustavo A. Hernandez-Fuentes, Fabian Rojas-Larios, Carmen A. Sanchez-Ramirez, Margarita L. Martinez-Fierro, Iram P. Rodriguez-Sanchez, José Guzmán-Esquivel, Idalia Garza-Veloz, José E. Del-Río-Valdivia, Jorge E. Plata-Florenzano, Iván Delgado-Enciso

**Affiliations:** 1 School of Medicine, University of Colima, Colima 28040, Mexico; fuentes_murguia@ucol.mx (M.F.-M.); 1933osiris@gmail.com (O.G.D.-E.); gahfuentes@gmail.com (G.A.H.-F.); frojas@ucol.mx (F.R.-L.); carmen_sanchez@ucol.mx (C.A.S.-R.); delriojose@ucol.mx (J.E.D.-R.-V.); 2 Cancerology State Institute, Colima State Health Services, Colima 28085, Mexico; plata_florenzano@hotmail.com; 3 Institute for Social Security and Services for State Workers (ISSSTE), State Delegation of Colima, Colima 28017, Mexico; mechevic@hotmail.com; 4 Molecular Medicine Laboratory, Academic Unit of Human Medicine and Health Sciences, Autonomous University of Zacatecas, Zacatecas 98160, Mexico; margaritamf@uaz.edu.mx (M.L.M.-F.); idaliagv@uaz.edu.mx (I.G.-V.); 5 Molecular and Structural Physiology Laboratory, School of Biological Sciences, Autonomous University of Nuevo Leon, San Nicolas de los Garza 66455, Mexico; iramrodriguez@gmail.com; 6 Clinical Epidemiology Research Unit, Mexican Institute of Social Security (IMSS), Villa de Alvarez, Colima 29883, Mexico; jose.esquivel@imss.gob.mx; 7 Robert Stempel College of Public Health and Social Work, Florida International University, Miami, FL 33199, USA

**Keywords:** diabetes mellitus, functional impairment of the shoulder, rotator cuff tendon, adhesive capsulitis, ultrasonography, case–control study

## Abstract

Background/Objectives: Patients with diabetes have been reported to experience a higher prevalence of shoulder disorders compared to those without diabetes or with other medical conditions. However, the specific types of shoulder injuries and the extent of functional impairment associated with diabetes mellitus remain unclear. This study aimed to assess the association between diabetes and specific shoulder injuries, as well as the degree of functional impairment in affected patients. Methods: A case–control study was conducted involving 136 patients with shoulder functional impairment (UCLA Shoulder Scale ≤ 27). The study included 38 patients with diabetes and 98 non-diabetic controls. Shoulder injuries were diagnosed using ultrasonography, focusing on the supraspinatus tendon, long head of the biceps tendon, subscapularis tendon, and the presence of adhesive capsulitis or rotator cuff tears. Results: Diabetic patients had significantly higher rates of poor shoulder function compared to non-diabetic controls (89.47% vs. 63.26%, adjusted OR [adOR] 5.22, 95% CI 1.57–17.32, *p* = 0.007). While both groups had high rates of supraspinatus and long head of the biceps tendon injuries (~80%), no significant differences were found between them (*p* > 0.300). However, diabetic patients were more than three times as likely to have subscapularis tendon injuries (adOR 3.15, 95% CI 1.26–7.90, *p* = 0.014) and massive rotator cuff tears (adOR 3.76, 95% CI 1.16–12.15, *p* = 0.027). Additionally, diabetes was associated with a fourfold increased risk of adhesive capsulitis (adOR 4.16, 95% CI 1.20–14.47, *p* = 0.025). Conclusions: Diabetes mellitus is linked to greater functional and structural deterioration of the shoulder, highlighting the importance of considering diabetes as a risk factor for specific shoulder injuries. Early diagnosis and treatment may improve outcomes for diabetic patients with shoulder disorders.

## 1. Introduction

Diabetes mellitus (DM) is a chronic disease characterized by high concentrations of glucose in the blood, estimated to be one of the leading causes of combined death and disability in the world, with almost 537 million people suffering from it worldwide, according to estimates for the year 2021 [1,2]. In addition to its metabolic complications, such as cardiovascular disease and neuropathy, diabetes has been associated with a range of musculoskeletal disorders and other complications, which can further complicate disease management. For instance, diabetic patients often experience peripheral neuropathy, which can lead to decreased sensation and increased risk of foot ulcers and infections.

Additionally, they may develop diabetic retinopathy, resulting in vision impairment and further complications in daily activities. Furthermore, clinical and experimental studies have revealed that DM is associated with poor wound healing, reduced bone mass, deteriorated collagen production, increased stiffness, and an increased risk for tendinopathy, with a higher presence of advanced glycation end products (AGE) [3,4]. Additionally, DM is associated with various musculoskeletal disorders, with adhesive capsulitis (AC) and rotator cuff tendon injuries being the most commonly occurring lesions in the shoulder joint [5,6]. Furthermore, diabetes can lead to muscle complications, most commonly in diabetic patients who require insulin, such as sarcopenia, characterized by loss of muscle mass and strength, which further complicates rehabilitation efforts and functional recovery [7,8]. Moreover, it has been observed that following tendon surgical repair, diabetic patients have a higher risk of infection, an increased incidence of re-tears, and a restricted range of motion in the shoulder [9,10].

Studies have shown that diabetes patients have a higher prevalence rate of shoulder disorders (27.5%) than patients without diabetes (5.0%) with general medical disorders [11,12]. The complications associated with diabetes, such as reduced collagen production, poor wound healing, and increased stiffness, can contribute to the development of shoulder disorders [8].

It is important to emphasize that the presence of diabetes is not the only factor to consider; there are other factors associated with the diminished quality of life in DM patients. First and foremost, adherence to medication is crucial in managing diabetes effectively. Poor adherence can exacerbate complications and contribute to a higher incidence of musculoskeletal issues, leading to increased pain and decreased mobility [13,14]. Additionally, lifestyle factors such as diet, physical activity, and weight management significantly impact the overall well-being of individuals with diabetes [15].

Common interventions aimed at enhancing mobility in diabetic patients encompass physical therapy, customized exercise regimens, and the utilization of assistive devices like braces and orthotics to aid joint functionality and reduce pain [16,17]. The American Diabetes Association recommends that adults with diabetes engage in a minimum of 150 min of moderate-intensity aerobic physical activity (50–70% of maximum heart rate) each week, distributed over at least three days, ensuring that no more than two consecutive days are without exercise [18], but, given the sociodemographic (age, BMI, clinical history socioeconomic factors) conditions in most of these patients, there is a lack of this activity.

In patients who are both diabetic and elderly, multiple risk factors converge, including neuropathy, impaired circulation, and comorbidities that can exacerbate deterioration and complicate wound healing [19,20]. Studies indicate that older adults with diabetes are more likely to experience functional decline compared to their younger counterparts, with a significant percentage facing challenges related to mobility and daily activities [21]. For the diagnosis of shoulder injuries from a morphological standpoint, the gold standard is magnetic resonance imaging (MRI) [22]. However, currently, ultrasound is the most utilized method because it is effective, less costly, more accessible, dynamic, and even more conclusive than MRI [23,24,25]. It has been demonstrated that ultrasound has a diagnostic reliability index similar to that of magnetic resonance imaging (MRI), with a sensitivity and specificity of 90% [26]. Nevertheless, the morphological evaluation of the shoulder is complemented by functional assessment [26].

Various instruments are used for the functional assessment of the shoulder, such as the Simple Shoulder Test (SimpleST) [27], the Constant–Murley Score (CMS) [28], scores on the American Shoulder and Elbow Surgeons (ASES) [29], and The University of California—Los Angeles Shoulder Scale (UCLA-SS) [30], with the latter being validated and widely used. The UCLA-SS consists of 35 items that evaluate pain, function, active forward flexion, muscle strength in active forward flexion, and patient satisfaction [31]. This scale is particularly beneficial in providing a comprehensive understanding of the patient’s shoulder condition and enables clinicians to track changes in functional status over time, thus facilitating the assessment of treatment efficacy [32,33].

However, there are a few reports detailing the specific types of shoulder lesions associated with diabetes mellitus and the corresponding degree of functional impairment [7,15]. This study seeks to address this gap by exploring these connections, aiming to provide valuable insights into the impact of diabetes on shoulder health. Particularly, it focuses on older adult populations, who may face compounded risks due to age-related changes and the presence of diabetes.

## 2. Materials and Methods

### 2.1. Study Design and Ethics

A case–control study was conducted at the Shoulder Clinic of the ISSSTE “Dr. Miguel Trejo Ochoa” Hospital in Colima City, Mexico. The present study was carried out following the Declaration of Helsinki (2013) and was approved by the Research Committee of the Regional University Hospital of Colima City, Mexico, with registration number CI/2019/01/CR/MI/073, on 28 February 2019, and by the Ethics Committee in Research of the same hospital with registration number 2019/I/CR/CL/REH/145, on 23 May 2019. Informed consent was obtained from all patients to be included in the study. Participation of subjects was voluntary, and informed consent was obtained from each subject. Anonymity was guaranteed to all patients.

### 2.2. Inclusion Criteria

**Figure 1:**
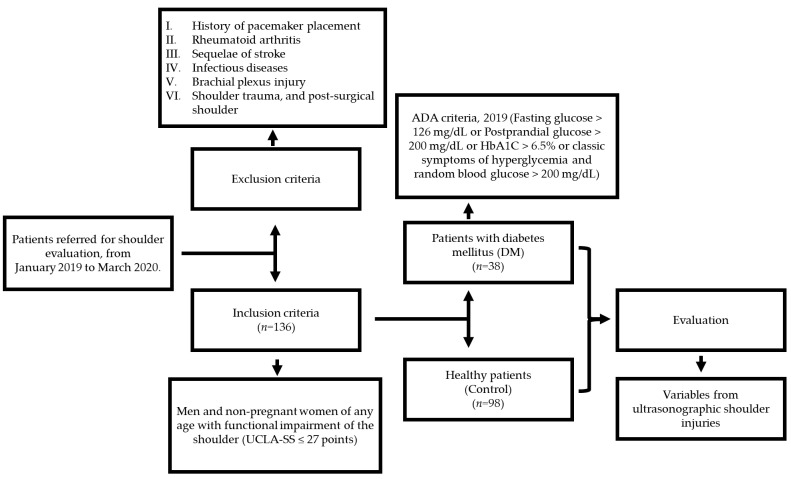
Flowchart of inclusion and exclusion criteria for the evaluation of functional shoulder impairment in patients with and without diabetes mellitus (control).

To be included in the study, subjects had to be insured at the public hospital “Dr. Miguel Trejo Ochoa”, part of the Institute for Social Security and Services for State Workers (ISSSTE) in Colima City, Mexico. Patients with functional shoulder impairment were initially treated by their family physician with standard conservative treatment (non-steroidal anti-inflammatory drugs, NSAIDs) for at least 3 months. Those who did not experience resolution of their symptoms were referred to the hospital’s “Shoulder Clinic”, where they were recruited according to the inclusion and exclusion criteria summarized in Figure 1.

The inclusion criteria involved men and non-pregnant women of any age with functional shoulder impairment lasting 3 months or more, as defined by the UCLA Shoulder Scale (UCLA-SS ≤ 27 points). Patients diagnosed with diabetes mellitus based on the 2019 ADA criteria were assigned to the case group, while those without diabetes were assigned to the control group. Patients with conditions such as pacemaker placement, rheumatoid arthritis, stroke sequelae, or a history of shoulder trauma were excluded from the study. Further details can be found in Figure 1.

The UCLA Shoulder Scale is a valuable tool for assessing patients with shoulder dysfunction, particularly related to degenerative joint disease. The latest version of the scale ranges from 0 (indicating maximum dysfunction) to 35 (indicating maximum comfort). Points are allocated as follows: 10 points for pain, 10 points for function, 5 points for active forward flexion, 5 points for strength in forward flexion, and 5 points for satisfaction. The scoring system categorizes results as follows: an excellent score ranges from 34 to 35 points, a good score from 28 to 33 points, a fair score (regular function) from 21 to 27 points, and a poor score (poor function) from 0 to 20 points [34].

Non-probabilistic consecutive sampling was used, including all patients referred to the “Shoulder Clinic” for shoulder evaluation who met the inclusion criteria, from January 2019 to March 2020. The sample size calculation was conducted to detect differences between the two proportions, using a prevalence rate of 27.5% for shoulder disorders in patients with diabetes compared to a rate of 5.0% in patients without diabetes [5]. The calculation was based on an alpha error of 0.05, a beta error of 0.2, an enrollment ratio of 2:1 (2 controls for every 1 case), and a statistical power of 0.8, which were performed with the online software ClinCalc version 1 (https://clincalc.com/stats/Power.aspx; accessed 30 January 2019) [35]. The result was a minimum sample size of 28 subjects in the case group and 56 in the control group. The final case and control groups included 38 and 98 subjects, respectively, exceeding the planned sample size. All patients underwent functional and ultrasound evaluation by two physicians to confirm the diagnosis, and in the case of diagnostic inconsistencies, a third evaluating physician determined the final ultrasound diagnosis. These physicians were blinded to the presence or absence of diabetes mellitus in the evaluated patients. Shoulder ultrasound evaluation was performed using a Philips^®^ HD11 XE ultrasound machine (Philips Healthcare, Bothell, WA, USA) with a linear transducer L12-3, according to a standard protocol previously described by Mantilla et al. [36].

The variables of interest for analysis were ultrasonographic shoulder injuries [supraspinatus tendon (SST) injury, infraspinatus tendon (IST) injury, subscapularis tendon (SBT) injury, massive rotator cuff tear, long head of biceps tendon (LHBT) injury, subacromial impingement syndrome (SIS), and bursitis (B)], functional shoulder impairment according to the (UCLA) Shoulder Scale, and the presence of adhesive capsulitis (AC).

The independent variable was diabetes mellitus while intervening variables included age, sex, and occupational activity. Occupational activities were classified according to the definitions established in the Code of Federal Regulations [37]. Specifically, light work encompasses jobs that require lifting no more than 20 pounds at a time, with frequent lifting or carrying of objects weighing up to 10 pounds. This category typically involves a considerable amount of walking or standing or entails sitting most of the time with some pushing and pulling of arm or leg controls. Common examples of light work include household tasks and office duties. In contrast, heavy work involves lifting no more than 100 pounds at a time, with frequent lifting or carrying of objects weighing up to 50 pounds. Jobs categorized as heavy work often necessitate substantial physical effort and may include activities such as farm work, factory labor, and construction tasks [37]. This classification is critical for understanding how varying levels of occupational activity may influence shoulder health among patients with diabetes mellitus [11,33]. It was also assessed whether the affected shoulder was considered dominant, defined as the one with which the patient had the greatest ability and was most used in their daily activities. Data were recorded, and each patient was informed of their results [33,37]. They were referred to a specialist physician at their healthcare unit for appropriate treatment. These methods were followed according to the STROBE Checklist for case–control studies, ensuring systematic consideration of variables and rigorous data collection procedures [38].

### 2.3. Statistical Analysis

The statistical analysis was conducted using descriptive statistics. All quantitative variables were dichotomized to transform them into qualitative variables and make comparisons between groups using the Chi-square test (χ^2^). The degree of association was determined using the odds ratios (OR), with a 95% confidence interval. The adjustment was made for the variables of sex (male/female) and age > 60 years (yes/no) using the Cochran–Mantel–Haenszel method. The IBM^®^ SPSS^®^ statistical package (version 25) was used for all analyses, except for the calculation of the sample size and statistical power, which were performed using CinCalc version 1 (https://clincalc.com/stats/Power.aspx; accessed 29 December 2023). A value of *p* < 0.05 was considered statistically significant.

## 3. Results

A total of 136 patients who met the inclusion criteria were included in the study. The demographic and clinical characteristics of the patients are shown in Table 1. No differences were observed between the groups regarding age, sex, occupational activity, dominant arm, or degrees of obesity. Female sex and light occupational activity predominated in both groups (see Table 1). In the diabetic group (cases), the time elapsed from the diagnosis of diabetes to the ultrasonographic analysis of the shoulder in this study was 5.32 ± 7.26 years.

The median UCLA-SS score in patients with functional impairment of the shoulder was 11.50 ± 5.03 (Q1–Q3: 8.0–15.0) and 16.5 ± 5.9 (Q1–Q3: 12.0–21.0) in patients with and without DM, respectively (*p* < 0.001). Poor shoulder function was observed in 89.47% (34/38) of patients with DM, compared to 63.26% (62/98) of patients in the control group. It was estimated that patients with DM have more than 5 times the odds of having poor shoulder function compared to patients without DM (AdOR 5.22, 95% CI 1.57–17.32, *p* = 0.007) (Table 2). At the end of the study, the statistical power for detecting a difference between groups was calculated (alpha = 0.05), utilizing the number of patients with poor shoulder function, and the result was 90.5%.

**Figure 2:**
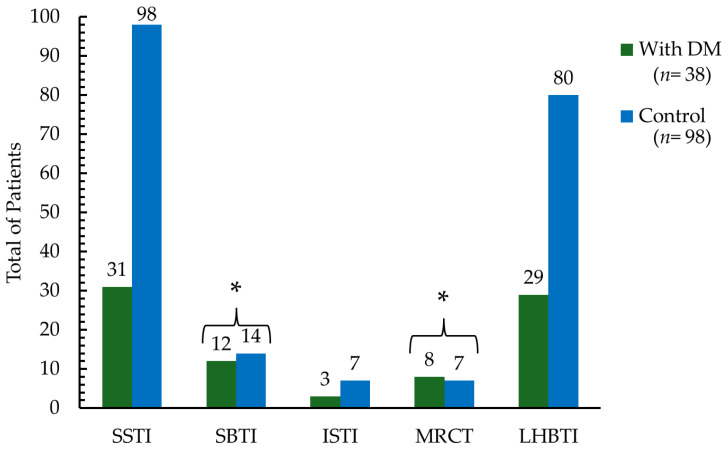
Distribution of Rotator Cuff Tendon Injuries (RCTIs) and Long Head of Biceps Tendon Injury (LHBTI) in patients with diabetes mellitus (DM) and controls. RCTI includes (1) supraspinatus tendon injury (SSTI), (2) subscapularis tendon injury (SBTI), (3) infraspinatus tendon injury (ISTI), and 4) massive rotator cuff tear (MRCT). * *p*-values were determined using the χ^2^ test. Statistically significant differences are indicated by asterisks (* *p* < 0.05).

The ultrasonographic findings of the shoulder with functional impairment are presented in Table 3 and Figure 2. Approximately 80% of the patients had supraspinatus tendon injury (SSTI) and/or long head of biceps tendon injury (LHBTI), with no significant differences found between patients with and without DM (see Table 3 and Figure 2). The least common injury was the infraspinatus tendon injury (ISTI), which was present in 7% of the patients, with no significant differences between patients with and without diabetes (*p* = 0.851). However, it was determined that patients with DM had over three times the likelihood of having a subscapularis tendon injury (SBTI) and massive rotator cuff tear (MRCT) compared to non-diabetics (see Table 3). Additionally, patients with diabetes also had a four times higher risk of adhesive capsulitis of the shoulder (AdOR 4.16, 95% CI 1.20–14.47, *p* = 0.025), observed in 18.42% and 5.10% of patients with and without DM, respectively. Post hoc power analyses for the association between specific ultrasonographic lesions (SBTI, MRCT, or adhesive capsulitis) and the presence of diabetes mellitus indicated a moderate statistical power, with values of 62.1%, 62.0%, and 65.7%, respectively [39,40]. The number of lesions (SSTI + SBTI + ISTI + MRCT + LHBTI) that each patient presented was 2 (Q1–Q3: 1.0–2.2) and 2 (Q1–Q3: 1.0–2.0) for patients with and without DM, respectively (*p* = 0.457).

## 4. Discussion

In patients with functional shoulder impairment, both functional and structural involvement detected by ultrasound are greater in diabetic patients compared to non-diabetic individuals. This finding is consistent with previous studies indicating that overall, function is reduced in patients with diabetes compared to healthy subjects [41]. It has been postulated that this phenomenon may be attributed to sustained hyperglycemia, which promotes the increase in advanced glycation end products (AGEs), altering the physicochemical properties of collagen and resulting in tissues that are more fragile and have reduced repair capacity [9,10,22,42,43].

This study also describes that AC was more probable in diabetic patients (AdOR 4.16, *p* = 0.025). This is similar to previous studies, which have reported that AC is a common alteration occurring in patients with diabetes mellitus [5,35]. From an ultrasonographic standpoint, the supraspinatus tendon (one of the tendons of the rotator cuff) was the most affected (in approximately 80% of patients with functional impairment). It has been previously postulated that this injury is common due to its location in the critical zone proximal to the greater tuberosity, where vascularity is poor and thus susceptible to degeneration and tears [4,44,45]. Another very common lesion (also in nearly 80% of patients) was the long head of biceps tendon injury (LHBTI), previously categorized as an initiating factor for shoulder disease [4,46]. Neither of these two previous lesions was associated with DM. On the other hand, subscapularis tendon injury (SBTI) was more frequent in patients with diabetes mellitus (DM). Mueller et al. (2016) had previously reported that DM is a risk factor for subscapular tendon injury [46]. In the present study, MRCT was associated with diabetes mellitus (DM), a finding not previously reported in the literature. This injury was observed in 21.05% of patients with DM, which aligns closely with the results of a population-based cohort study conducted by Shih-Wei Huang (2016) [47]. In this retrospective longitudinal study, which spanned seven years, Huang and colleagues examined the risk of rotator cuff repair surgery in a large cohort, comprising 58,652 patients with DM and 117,304 matched controls without DM (matched for age and sex). They reported a frequency of rotator cuff injuries of 28.2% in the diabetic cohort. Additionally, the incidence of rotator cuff repair surgery was notably higher among patients with DM, recorded at 41 per 100,000 person-years, compared to 26 per 100,000 person-years in the non-DM cohort [47]. This reinforces the association between DM and rotator cuff injuries, highlighting the increased risk faced by patients with diabetes.

In this study, descriptive statistics were used to summarize the participants’ demographic and clinical characteristics. Quantitative variables, such as age and BMI, were dichotomized to facilitate group comparisons. The Chi-square test (χ^2^), a non-parametric test suitable for categorical data, was employed to assess differences between diabetic (DM) and non-diabetic patients in terms of shoulder function and ultrasound-detected injuries. *p*-values below 0.05 were considered statistically significant. Odds ratios (ORs) were calculated to quantify the association between DM and outcomes like poor shoulder function and specific tendon injuries. The adjusted odds ratios (AdORs), refined through the Cochran–Mantel–Haenszel method, accounted for confounders such as sex and age (>60 years), providing a more accurate estimate of the associations. Compared to previous studies that employed similar methodologies, such as Jayaswal et al. (2021), which examine hypertension and mortality by COVID-19 in a non-diabetic population and diabetic population [48,49], our approach focused specifically on the diabetic group, adjusting for key confounding factors, offering a more tailored insight into the relationship between DM and shoulder disfunction. The use of adjusted odds ratios and the Cochran–Mantel–Haenszel method ensured that potential confounders such as sex and age were accounted for, enhancing the reliability of the findings [50].

In our study, we recognize the need to strengthen the distinction between light and heavy occupational activities, particularly in relation to their impact on shoulder health [37]. Light occupational activities, such as household and office work, may present different risk factors for shoulder pathologies compared to heavy activities like farm work or construction. In the present study, the categories of occupational activity (light and heavy) did not differ between patients with and without DM (see Table 1). However, the study also had limitations. The relatively small sample size, while adequate for detecting significant associations, may limit the generalizability of the findings. Moreover, certain factors, such as the influence of medications (e.g., insulin, oral hypoglycemics) on musculoskeletal health, were not considered in the statistical models, potentially acting as confounding factors. While our analysis adjusted for sex and age as intervening variables, we did not include occupational activity in the final statistical adjustments. This decision was influenced by the complexity of categorizing occupational activity into broad classifications, such as light versus heavy work. Such a categorization may not fully capture the specific types of tasks or the intensity of physical demands that can impact shoulder health. Furthermore, it is essential to consider the role of sporting activities in this context. Engaging in sports may not only affect shoulder performance but can also be influenced by the presence of chronic conditions. Research has shown that rehabilitation outcomes in athletes can vary based on their underlying health status and activity levels. For example, studies by Farì et al. (2022) and (2023) highlight the biomechanics of shoulder pain and the effectiveness of rehabilitation strategies in athletes, specifically in wheelchair basketball players [51,52].

Additionally, our sample size constraints played a significant role in this decision. Adjusting multiple variables, including occupational activity, could have compromised the statistical power of our analysis. As a result, we prioritized the adjustment for age and sex, which are well-documented confounders in research related to musculoskeletal health. We acknowledge the potential influence of occupational activity on musculoskeletal conditions and plan to explore this aspect in future studies. By employing larger sample sizes and conducting more detailed assessments of occupational exposure, we aim to provide a more comprehensive understanding of how this variable may affect shoulder injuries in the context of diabetes mellitus.

Certain other limitations should also be acknowledged. Medications such as insulin or oral hypoglycemics, which could influence musculoskeletal health or healing processes, were not considered. These treatments may act as confounding factors in the association between DM and shoulder injuries [53]. Additionally, other potential confounders, such as the patient’s diet or the use of alternative or complementary treatments, such as herbal medicine, were not explored in this study. Including these factors could provide a more precise analysis of the impact of DM-related treatments and lifestyle choices on musculoskeletal conditions.

In the future, it would be valuable to explore these aspects and establish connections between DM and other comorbidities to potentially extrapolate these results to broader populations and other related health conditions [53]. Additionally, only the UCLA Shoulder Scale was used to evaluate shoulder function. The inclusion of other validated tools, such as the Constant–Murley Score (CMS) or the American Shoulder and Elbow Surgeons (ASES) scale, could have offered a broader perspective on shoulder function and impairment, thereby strengthening the analysis of DM’s overall impact on shoulder health [54]. Future research should also consider additional factors, such as osteodegenerative changes, long-term glycemic control, and the duration of diabetes, to better understand their contributions to shoulder pathologies in diabetic patients.

Among the strengths of the study is the use of ultrasound as a diagnostic tool. Ultrasound offers a non-invasive, dynamic, and cost-effective method for detecting shoulder injuries, making it a practical and accessible option in clinical settings. Furthermore, a detailed functional assessment was conducted, allowing for the evaluation of both morphological and functional aspects of the injuries, providing a more holistic view of DM’s impact on shoulder health. The study’s high statistical power of 90% ensured the reliable detection of differences between diabetic and non-diabetic groups, particularly concerning functional impairments such as subscapularis tendon injuries and massive rotator cuff tears. The use of adjusted odds ratios (AdOR) for age and sex helped mitigate potential biases, reinforcing the validity of the association between DM and specific shoulder injuries. However, a limitation of the study was that post hoc power analyses for the association between specific ultrasonographic lesions and diabetes mellitus indicated a moderate statistical power (62–65%). Therefore, future studies with a larger number of patients are needed to confirm these results more robustly.

Finally, it is important to point out that while our study primarily focused on the association between diabetes mellitus and shoulder injuries, several additional factors, such as the presence of a downward-sloping type III acromion, mild to moderate acromioclavicular degenerative changes, and the acromiohumeral distance, can significantly influence the development of shoulder pathologies [55,56,57]. Acknowledging these variables in future research will enhance our understanding of the complex mechanisms underlying shoulder dysfunction in diabetic patients. Incorporating these factors into our analyses can lead to more comprehensive insights and inform targeted interventions aimed at improving clinical outcomes for this population. This consideration not only addresses the potential limitations of our current study but also paves the way for exploring the multifaceted relationship between diabetes and shoulder health in greater depth.

In terms of clinical relevance, the study’s findings provide crucial insights into the higher risk of shoulder impairments in diabetic patients, which can lead to more effective preventive measures and timely interventions to improve the management of shoulder pathologies in this population. However, further exploration of factors such as physical activity levels or occupation type would have been beneficial, as these may interact with DM to influence injury risk. Future research could also include biomarkers, such as advanced glycation end products (AGEs), to provide a biochemical correlation between DM and musculoskeletal changes, offering a more comprehensive understanding of the underlying mechanisms contributing to shoulder pathologies in diabetic patients [58,59].

An important aspect to mention is that the time elapsed from diabetes diagnosis to the development of shoulder lesions reported in this study was 5.32 ± 7.26 years. However, diabetes diagnosis in Mexico may often be delayed [60], which suggests this period could be longer than reported. Additionally, a previous study conducted in Mexico found that only 48.6% of diabetic patients who regularly attend consultations achieve adequate glycemic control [61]. Consequently, the duration of diabetes and the degree of glycemic control are important factors that should be studied in greater detail in future research on shoulder or other joint lesions.

## 5. Conclusions

In conclusion, diabetes mellitus was significantly associated with greater functional impairment of the shoulder, characterized by a higher prevalence of subscapularis tendon injuries, massive rotator cuff tears, and adhesive capsulitis. These findings underscore the importance of recognizing DM as a key risk factor for shoulder injuries, which could enhance preventive strategies and facilitate earlier diagnosis and targeted treatment. Addressing these risks proactively may improve patient outcomes, highlighting the need for clinicians to integrate regular musculoskeletal assessments in diabetic care protocols to mitigate the progression of shoulder-related complications.

## Figures and Tables

**Table 1:** Demographic and clinical characteristics of studied patients.

Variable	Groups *n* = 136 (100%)		
	With DM *n* = 38 (27.94%)	Control *n* = 98 (72.05%)	*p* * Value
Age (years)	58.95 (SD 8.47)	60.33 (SD 12.29)	0.527
Sex			0.508
Male	20.51% (8/38)	26.53% (26/98)	
Female	78.94% (30/38)	73.46% (72/98)	
BMI (kg/m^2^)			0.073
Underweight < 18.4	5.26% (2/38)	2.04% (2/98)	
Normal weight 18.5–24.9	10.52% (4/38)	10.20% (10/98)	
Overweight 25–29.9	34.21% (13/38)	39.79% (39/98)	
Obesity I 30–34.9	18.42% (7/38)	32.65% (32/98)	
Obesity II 35–39.9	26.31% (10/38)	15.30% (15/98)	
Obesity III >40	5.26% (2/38)	0% (0/98)	
Affected shoulder			
Dominant	65.78% (25/38)	63.265% (62/98)	0.277
Non-dominant	34.21% (13/38)	36.73% (36/98)	0.196
Occupational activity			0.338
Light	86.84% (33/38)	77.55% (76/98)	
Heavy	13.15% (5/38)	22.44% (22/98)	

SD, (Standard deviation). BMI (Body Mass Index). kg/m^2^ (kilograms per square meter). * *p*-values were calculated using the χ^2^ test.

**Table 2:** Functional Impairment of the Shoulder—UCLA Shoulder Scale.

Groups *n* = 136 (100%)	Functional Impairment of the Shoulder		AdOR (CI 95%)	*p* * Value
	Regular Function	Poor Function		
			5.22 (1.57–17.32)	0.007
DM (*n* = 38)	10.52% (4/38)	89.47% (34/38)		
Without DM (*n* = 98)	32.65% (32/98)	63.26% (62/98)		

AdOR represents the adjusted odds ratio, which has been adjusted for the variables of sex (male/female) and age > 60 years (yes/no) using the Cochran–Mantel–Haenszel method. DM stands for Diabetes Mellitus. Regular function: 21 to 27 points in the UCLA Shoulder Scale. Poor function: 0 to 20 points in the UCLA Shoulder Scale. * *p*-values were calculated using the χ^2^ test.

**Table 3:** Ultrasonographic shoulder injuries in patients with and without diabetes mellitus (DM).

Injuries	Groups *n* = 136 (100%)		AdOR	CI 95%	*p* * Value
	With DM *n* = 38 (27.94%)	Control *n* = 98 (72.05%)			
SSTI	81.57% (31/38)	79.59% (78/98)	1.21	0.44–3.33	0.701
SBTI	31.57% (12/38)	14.28% (14/98)	3.15	1.26–7.90	0.014
ISTI	7.89% (3/38)	7.14% (7/98)	1.14	0.27–4.89	0.851
MRCT	21.05% (8/38)	7.14% (7/98)	3.76	1.16–12.15	0.027
LHBTI	76.31% (29/38)	81.63% (80/98)	0.63	0.24–1.60	0.332

* *p*-values were calculated using the χ^2^ test. AdOR: Adjusted odds ratio. Adjusted for the variables of sex (male/female) and age > 60 years (yes/no) through the Cochran–Mantel–Haenszel method. The table describes Rotator Cuff Tendon Injuries (RCTIs) including (1) supraspinatus tendon injury (SSTI), (2) subscapularis tendon injury (SBTI), (3) infraspinatus tendon injury (ISTI), and (4) massive rotator cuff tear (MRCT). Long head of biceps tendon injury (LHBTI) is also described.

## Data Availability

The original contributions presented in the study are included in the article; further inquiries can be directed to the corresponding authors.
